# Regional effects of streptozotocin‐induced diabetes on shortening and calcium transport in epicardial and endocardial myocytes from rat left ventricle

**DOI:** 10.14814/phy2.13034

**Published:** 2016-11-24

**Authors:** Manal M. A. Smail, Muhammad A. Qureshi, Anatoliy Shmygol, Murat Oz, Jaipaul Singh, Vadym Sydorenko, Alya Arabi, Frank C. Howarth, Lina Al Kury

**Affiliations:** ^1^ Department of PhysiologyCollege of Medicine & Health SciencesUAE UniversityAl AinUAE; ^2^ Department of PharmacologyCollege of Medicine & Health SciencesUAE UniversityAl AinUAE; ^3^ School of Forensic & Applied SciencesUniversity of Central LancashirePrestonUK; ^4^ Department of Cellular MembranologyBogomoletz Institute of PhysiologyKievUkraine; ^5^ College of Natural & Health SciencesZayed UniversityAbu DhabiUAE

**Keywords:** Calcium transport, diabetes, endocardial myocytes, epicardial myocytes, rat heart, shortening, streptozotocin, ventricle

## Abstract

In the heart, the left ventricle pumps blood at higher pressure than the right ventricle. Within the left ventricle, the electromechanical properties of ventricular cardiac myocytes vary transmurally and this may be related to the gradients of stress and strain experienced in vivo across the ventricular wall. Diabetes is also associated with alterations in hemodynamic function. The aim of this study was to investigate shortening and Ca^2+^ transport in epicardial (EPI) and endocardial (ENDO) left ventricular myocytes in the streptozotocin (STZ)‐induced diabetic rat. Shortening, intracellular Ca^2+^ and L‐type Ca^2+^ current (*I*_Ca,L_) were measured by video detection, fura‐2 microfluorimetry, and whole‐cell patch clamp techniques, respectively. Time to peak (TPK) shortening was prolonged to similar extents in ENDO and EPI myocytes from STZ‐treated rats compared to ENDO and EPI myocytes from controls. Time to half (THALF) relaxation of shortening was prolonged in ENDO myocytes from STZ‐treated rats compared to ENDO controls. TPK Ca^2+^ transient was prolonged in ENDO myocytes from STZ‐treated rats compared to ENDO controls. THALF decay of the Ca^2+^ transient was prolonged in ENDO myocytes from STZ‐treated rats compared to ENDO controls. Sarcoplasmic reticulum (SR) fractional release of Ca^2+^ was reduced in EPI myocytes from STZ‐treated rats compared to EPI controls. *I*_C__a,L_ activation, inactivation, and recovery from inactivation were not significantly altered in EPI and ENDO myocytes from STZ‐treated rats or controls. Regional differences in Ca^2+^ transport may partly underlie differences in ventricular myocyte shortening across the wall of the healthy and the STZ‐treated rat left ventricle.

## Introduction

Blood is pumped at a higher pressure in the left ventricle compared to the right ventricle. Within the left ventricle, the electromechanical properties of ventricular cardiac myocytes vary transmurally (Campbell et al. 2008, 2009; Vasil'eva and Solov'eva 2012). This may be related to the gradients of stress and strain experienced in vivo across the ventricular wall. Electrophysiological heterogeneity across the ventricular wall is a result of differential transmural expression of various ion channel proteins that underlie the different action potential waveforms observed in epicardial (EPI), midmyocardial (MID), and endocardial (ENDO) regions. White et al. (1988) reported that the cross‐sectional area of ENDO myocytes is greater than in EPI myocytes and exercise training increases the size of myocytes in EPI but not ENDO regions in rat heart. De Clerck et al. (1984) reported no significant difference in resting cell dimensions and sarcomere length between cells isolated from the left and right ventricle nor between cells from EPI or ENDO regions of rat heart. Anversa et al. (1978) reported that ENDO regions of the left ventricular myocardium contain 30% more myocytes, 27% less interstitial space, 48% less capillary volume, 17% less capillary surface, and the same capillary length per unit tissue volume compared to EPI rat myocytes. Various diseases can alter the ultrastructure of the heart. For example, 14 weeks of hypertension increased left ventricular weight by 30%, wall thickness by 42%, while the number of myocytes and total length of capillaries remained constant. The EPI region enlarged by 37% with proportional increases of myocyte and interstitial volumes. The ENDO enlargement was only 26%, composed of 21% hypertrophy of myocytes and 55% increase in interstitial component. Hypertrophy of myocytes was 76% greater in the EPI region and was accompanied by a reduced mitochondria to myofibril ratio (Anversa et al. 1978). In vivo echocardiography has confirmed that subendocardial layers contract more and faster than EPI layers of rat ventricular myocardium (Ait et al. 2009). McCrossan et al. (2004) reported that EPI, MID, and ENDO myocytes from left ventricle have similar volume. Cell lengths were longer in EPI and MID compared to ENDO and cell widths are similar in EPI, MID, and ENDO and length/width is larger in EPI compared to ENDO myocytes. Myocyte amplitude, time to peak (TPK) shortening, and time to half (THALF) relaxation of shortening were similar in EPI, MID, and ENDO myocytes (McCrossan et al. 2004). Natali et al. (2002) reported that length, width, depth, length to width ratio, volume and amplitude of shortening were similar in ENDO and EPI myocytes. In length‐tension experiments, ENDO myocytes from both ferret and rat ventricle were stiffer (i.e., had steeper sarcomere length–passive tension relationships) than EPI cells (Cazorla et al. 2000). Stones et al. (2007) reported no difference in inotropic response between rat EPI and ENDO left ventricular myocytes to axial stretch. When rat ventricle was stretched by inflation of an intraventricular balloon action potential, shortening was more pronounced in the endocardium compared with that of the epicardium (Kelly et al. 2006). The duration of action potential, using various measures including 25%, 75%, and 90% repolarization, was longer in ENDO compared to EPI myocytes (Shipsey et al. 1997; Volk et al. 1999, 2005; Natali et al. 2002; McCrossan et al. 2004; Wang et al. 2010). Differences in action potential duration have been partly attributed to changes in repolarizing ionic currents. For example, in rat heart, the capacitance was unaltered despite the reduction in transient outward (*I*_to_) and delayed rectifier (*I*_K_) and increase in the inward rectifier (*I*_K1_) in ENDO compared to EPI rat myocytes (Shimoni et al. 1995; Bryant et al. 1999; Volk et al. 1999; Yao et al. 1999; Wang et al. 2010). Mathematical modeling suggests that a smaller density and slower reactivation kinetics of *I*_to_ in ENDO myocytes may account for the longer action potential duration (Pandit et al. 2001). In general, *I*_Ca,L_, the primary trigger for SR Ca^2+^ release, appears to be similar in rat ventricular myocytes from ENDO compared to EPI regions (Bryant et al. 1999; Volk et al. 1999; Volk and Ehmke 2002). Some studies have reported a larger amplitude of the Ca^2+^ transient and prolonged TPK Ca^2+^ transient and a larger amplitude of caffeine‐evoked Ca^2+^ transient in rat ENDO compared to EPI ventricular myocytes (Fowler et al. 2005), while other studies have reported similar amplitude of the Ca^2+^ transient, TPK and THALF decay of the Ca^2+^ transient in EPI and ENDO myocytes (Natali et al. 2002; McCrossan et al. 2004). Aging may be a factor in the variability of results. Decay time of the Ca^2+^ transient and the time required for 50% length relaxation increased with age, but not uniformly across the EPI, MID, and ENDO rat ventricular myocytes (Haynes et al. 2014). Mathematical modeling suggests that the prolonged action potential duration in ENDO myocytes is consistent with an enhanced amplitude of the sustained K^+^ current (*I*_ss_) and larger influx of Ca^2+^ ions via L‐type Ca^2+^ channels, the latter resulting in increased loading of the SR (Pandit et al. 2001). Little is known about the transmural effects of diabetes mellitus on ventricular myocyte contraction and calcium transport. However, experiments in STZ‐induced diabetic rat in myocytes isolated from whole ventricle, have variously reported alterations in the amplitude and time course of shortening, which have been partly attributed to defects in Ca^2+^ transport including L‐type Ca^2+^ current, SR Ca^2+^ uptake and release, and Na^+^/Ca^2+^ exchange (Choi et al. 2002; Howarth et al. 2002; Bracken et al. 2006). The general aim of this project was to investigate the regional effects of streptozotocin (STZ)‐induced diabetes on shortening and calcium transport in EPI and ENDO from the left ventricle of rat heart.

## Methods

### Experimental model

Experiments were performed in STZ‐induced diabetic rats, a well‐characterized experimental model of diabetes mellitus (Howarth et al. 2002; Hamouda et al. 2015). Diabetes was induced in male (250 g) Wistar rats by a single intraperitoneal injection of STZ (60 mg/kg bodyweight) as previously described (Howarth et al. 2002; Hamouda et al. 2015). Bodyweight, nonfasting blood glucose, and heart weight were measured prior to the experiments. Experiments were performed in EPI and ENDO myocytes from left ventricle of STZ and control rats 3 months after treatment. All experiments were performed with local Ethics Committee approval, UAE University, Al Ain.

### Isolation of ventricular myocytes

Ventricular myocytes were isolated according to modifications of previously described techniques (Howarth et al. 2002; Hamouda et al. 2015). In brief, animals were killed using a guillotine and hearts were removed rapidly and mounted for retrograde perfusion on a Langendorff system. Hearts were perfused at a constant flow rate of 8 ml.g heart^‐1^.min^‐1^ and at 36–37°C with cell isolation solution containing in mmol/L: 130.0 NaCl, 5.4 KCl, 1.4 MgCl_2_, 0.75 CaCl_2_, 0.4 NaH_2_PO_4_, 5.0 HEPES, 10.0 glucose, 20.0 taurine, and 10.0 creatine (pH 7.3). When heart contraction had stabilized, perfusion was switched for 4 min to Ca^2+^‐free cell isolation solution containing 0.1 mmol/L EGTA, and then for 6 min to cell isolation solution containing 0.05 mmol/L Ca^2+^, 0.60 mg/mL type 1 collagenase (Worthington Biochemical Corp, Lakewood, NJ), and 0.075 mg/mL type XIV protease (Sigma, Taufkirchen, Germany). Left ventricle tissue was excised from the heart; a section of tissue was carefully dissected from ENDO and EPI regions, minced, and gently shaken in collagenase‐containing isolation solution supplemented with 1% BSA. Cells were filtered from this solution at 4‐min intervals and resuspended in cell isolation solution containing 0.75 mmol/L Ca^2+^.

### Ventricular myocyte shortening

Ventricular myocytes were isolated and shortening measured according to modifications of previously described techniques (Howarth et al. 2002; Hamouda et al. 2015). In brief, cells were superfused (3–5 mL/min) with normal Tyrode containing the following in mmol/L: 140.0 NaCl, 5.0 KCl, 1.0 MgCl_2_, 10.0 glucose, 5.0 HEPES, 1.8 CaCl_2_ (pH 7.4). Unloaded EPI and ENDO myocyte shortening were recorded using a video edge detection system (VED‐114; Crystal Biotech, Northborough, MA). Resting cell length, TPK shortening, THALF relaxation, and amplitude of shortening (expressed as a % of resting cell length) were measured in electrically stimulated (1 Hz) myocytes maintained at 35–36°C. Data were acquired and analyzed with Signal Averager software v 6.37 (Cambridge Electronic Design, Cambridge, UK).

### Intracellular Ca^2+^

Myocyte fura‐2 ratio (intracellular Ca^2+^) was measured in fura‐2 AM‐loaded myocytes according to previously described techniques (Howarth et al. 2002; Hamouda et al. 2015). In brief, cells were alternately illuminated by 340 nm and 380 nm light using a monochromator (Cairn Research, Faversham, UK) which changed the excitation light every 2 ms. The resulting fluorescence, emitted at 510 nm, was recorded by a photomultiplier tube and the ratio of the emitted fluorescence at the two excitation wavelengths (340/380 ratio) provided an index of intracellular Ca^2+^ concentration. Resting fura‐2 ratio, TPK Ca^2+^ transient, THALF decay of the Ca^2+^ transient, and the amplitude of the Ca^2+^ transient were measured in electrically stimulated (1 Hz) myocytes maintained at 35–36°C. Data were acquired and analyzed with Signal Averager software v 6.37 (Cambridge Electronic Design).

### Measurement of sarcoplasmic reticulum Ca^2+^ transport

SR Ca^2+^ was assessed using previously described techniques (Howarth et al. 2002; Hamouda et al. 2015). The protocol employed in this study is illustrated in Figure 3A. In brief, after establishing steady‐state Ca^2+^ transients in electrically stimulated (1 Hz) myocytes maintained at 35–36°C and loaded with fura‐2 AM, stimulation was paused for a period of 5 sec. Caffeine (20 mmol/L) was then applied for 10 sec using a solution‐switching device customized for rapid solution exchange (Levi et al. 1996). Electrical stimulation was then resumed and the Ca^2+^ transients were allowed to recover to steady state. Fractional release of SR Ca^2+^ was calculated by comparing the amplitude of the electrically evoked steady‐state Ca^2+^ transients with that of the caffeine‐evoked Ca^2+^ transient. Ca^2+^ refilling of the SR was assessed by measuring the rate of recovery of electrically evoked Ca^2+^ transients following application of caffeine.

### Measurement of L‐type Ca^2+^ current

Voltage‐dependent L‐type Ca^2+^ current was measured using whole‐cell patch clamp according to previously described techniques (Hamouda et al. 2015). In brief, *I*_Ca,L_ was recorded with an Axopatch 200B amplifier (Molecular Devices, Sunnyvale, CA). The analog signal was filtered using a four‐pole Bessel filter with a bandwidth of 5 kHz and digitized at a sampling rate of 10 kHz under software control (PClamp 10.6.2.2; Molecular Devices). Patch pipettes were fabricated from filamented BF150‐86‐10 borosilicate glass (Sutter Instrument, Novato, CA). The whole‐cell bath solution contained the following in mmol/L: 145 NaCl, 2 MgCl_2_, 2 CaCl_2_, 10 HEPES and 10 glucose (pH 7.35). The pipette solution contained the following in mmol/L: 140 CsCl, 2 MgCl_2_, 10 TEA Cl, 10 EGTA, 10 HEPES, 1 MgATP (pH 7.25). Electrode resistances ranged from 3 to 5 MΩ, and seal resistances were 1–5 GΩ. Series resistances were compensated to >75% of the uncompensated value. Experiments were performed at 34–36°C. The current–voltage relationship was obtained by applying 300‐ms test pulses in the range of −60 mV to +70 mV in 10 mV steps from a holding potential of −50 mV. The steady‐state inactivation of Ca^2+^ current was measured as the relationship of amplitude of the peak current at a test pulse of 0 mV to amplitude of peak current during 1000‐ms long prepulses to various voltages between −60 and +30 mV. Normalized peak currents measured after these prepulses were plotted against the respective prepulse potential. Time course of recovery from inactivation was measured using a two‐pulse protocol. Two 100‐ms depolarizing pulses to +10 mV were separated by interpulse intervals with variable duration. Peak Ca^2+^ current amplitude measured by the second pulse was normalized to that measured by the first pulse and their ratio was plotted against the interpulse interval. Data were acquired and analyzed with pClamp software v 10.6.2.2 (Molecular Devices).

### Statistics

The results were expressed as the mean ± SEM of “*n*” observations. Statistical comparisons were performed using one‐way ANOVA followed by Bonferroni‐corrected *t*‐tests for multiple comparisons. Null hypothesis was rejected at *P* < 0.05.

## Results

### General characteristics

The general characteristics of STZ‐treated rats compared to age‐matched controls are shown in Table 1. STZ‐treated rats displayed significantly (*P* < 0.01) lower bodyweight, heart weight, increased bodyweight/heart weight ratio, and a fivefold increase in nonfasting blood glucose compared to controls.

**Table 1:** General characteristics of streptozotocin‐treated rats

	CON	STZ
Bodyweight (g)	361.5 ± 5.9 (20)	255.8 ± 9.3 (23)^a^
Heart weight (g)	1.13 ± 0.01 (20)	0.96 ± 0.02 (23)^a^
Bodyweight/heart weight (mg/g)	3.13 ± 0.05 (20)	3.78 ± 0.09 (23)^a^
Blood glucose (mg/dL)	90.5 ± 1.7 (20)	455.0 ± 15.9 (23)^a^

Data are mean ± SEM, number of hearts is in parenthesis.

CON = Control

a *P* < 0.01.

### Ventricular myocyte shortening

**Figure 1:**
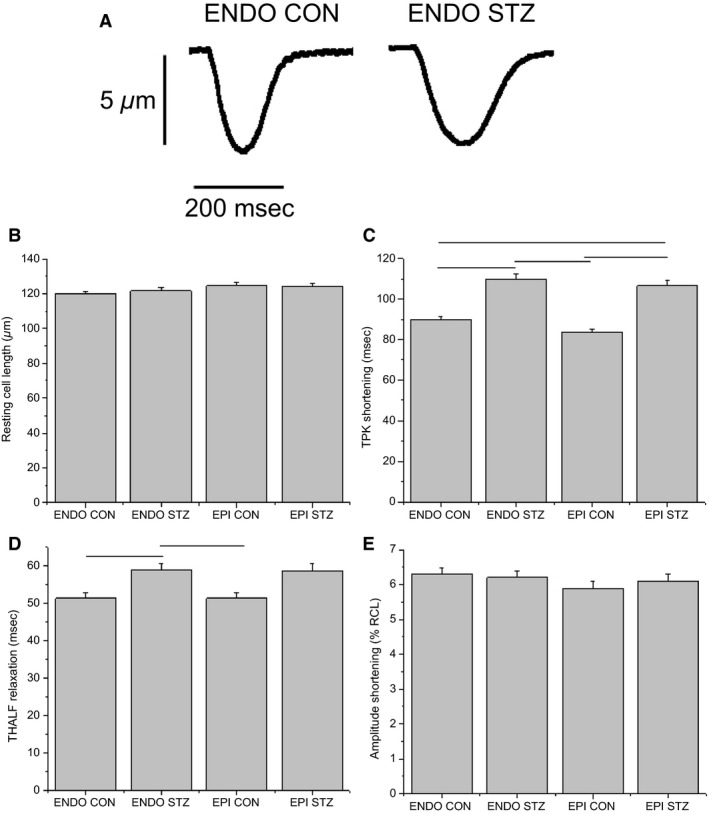
Ventricular myocyte shortening. Typical records of shortening in endocardial (ENDO) myocytes from streptozotocin (STZ)‐treated and control rat heart are shown in (A). Bar graphs showing the mean resting cell length (B), time to peak (TPK) shortening (C), time to half (THALF) relaxation of shortening (D), and amplitude of shortening (E). Data are mean + SEM., *n* = 109–126 cells from 20 to 23 hearts. Horizontal lines above the bars represent significant differences at the level of *P* < 0.05.

Typical records of shortening in ENDO myocytes from STZ‐treated and control rat heart are shown in Figure 1A. Resting cell length was not significantly (*P* > 0.05) altered in EPI and ENDO myocytes from STZ‐treated rats or controls (Fig. 1B). TPK shortening was not significantly altered in EPI compared to ENDO myocytes from STZ‐treated rats and controls. However, TPK was significantly (*P* < 0.05) prolonged, and to similar extents, in ENDO (110.0 ± 2.4 ms, *n* = 120 cells, 23 hearts) and EPI (106.7 ± 2.4 ms, *n* = 109 cells, 23 hearts) myocytes from STZ‐treated rats compared to ENDO (89.7 ± 1.6 ms, *n* = 126 cells, 20 hearts) and EPI (83.4 ± 1.9 ms, *n* = 119 cells, 20 hearts) myocytes from controls (Fig. 1C). THALF relaxation of shortening was not significantly altered in EPI compared to ENDO myocytes from STZ‐treated rats and controls. However, THALF relaxation of shortening was significantly prolonged in ENDO myocytes from STZ‐treated rats (58.9 ± 1.8 ms, *n* = 121 cells, 23 hearts) compared to controls (51.3 ± 1.4 ms, *n* = 126 cells, 20 hearts). There was also a modest prolongation of THALF relaxation in EPI myocytes from STZ‐treated rats compared to controls (Fig. 1D). Amplitude of shortening was not significantly (*P* > 0.05) altered in EPI and ENDO myocytes from STZ‐treated rats or controls (Fig. 1E). Analysis of shortening data in EPI and ENDO myocytes from a typical STZ and control heart showed the same trend as collective data presented in Figure 1A–E including unaltered RCL and amplitude of shortening, prolonged TPK in ENDO (106 ± 4 ms, *n* = 5) and EPI (110 ± 8 ms, *n* = 6) myocytes from STZ compared to ENDO (84 ± 3 ms, *n* = 13) and EPI (95 ± 6 ms, *n* = 9) myocytes from control heart, and prolonged THALF in ENDO (59 ± 8 ms, *n* = 6) myocytes from STZ compared to ENDO (52 ± 4, *n* = 7) myocytes from control heart. Experiments were then conducted to investigate whether alterations in Ca^2+^ transport may partly underlie the differences in shortening between ENDO STZ and ENDO control and between EPI STZ and EPI control myocytes.

### Intracellular Ca^2+^

**Figure 2:**
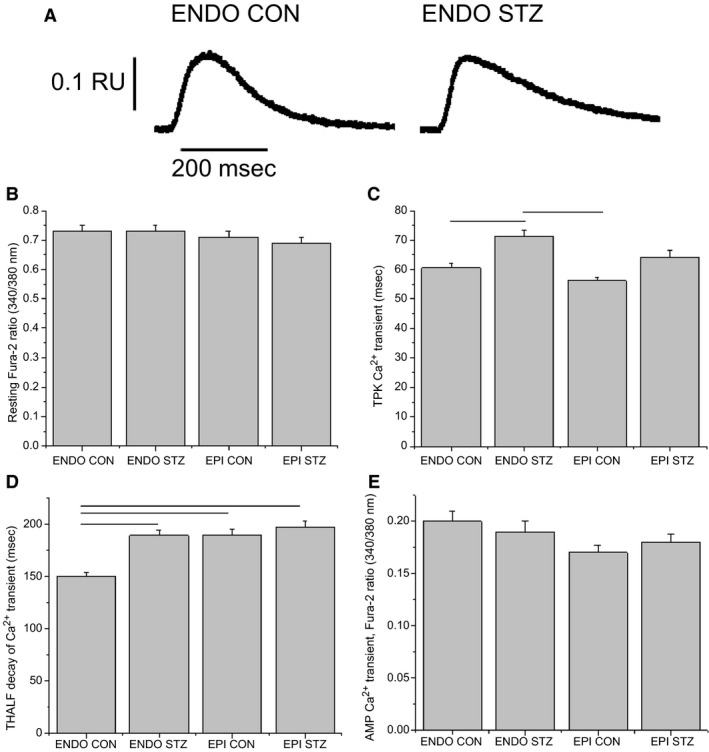
Ventricular myocyte Ca^2+^ transient. Typical records of Ca^2+^ transients in endocardial (ENDO) myocytes from streptozotocin (STZ)‐treated and control rat heart are shown in (A). Bar graphs showing the mean resting fura‐2 ratio (B), time to peak (TPK) Ca^2+^ transient (C), time to half (THALF) decay of the Ca^2+^ transient (D), and amplitude of the Ca^2+^ transient (E). Data are mean + SEM,* n* = 107–122 cells from 20 to 23 hearts. Horizontal lines above the bars represent significant differences at the level of *P* < 0.05.

Typical records of Ca^2+^ transients in ENDO myocytes from STZ‐treated and control rat heart are shown in Figure 2A. Resting fura‐2 ratio was not significantly (*P* > 0.05) altered in EPI and ENDO myocytes from STZ‐treated rats or controls (Fig. 2B). TPK Ca^2+^ transient was not significantly altered in EPI compared to ENDO myocytes from STZ‐treated rats and controls. However, TPK was significantly (*P* < 0.05) prolonged in ENDO (71.3 ± 2.2 ms, *n* = 113 cells, 23 hearts) myocytes from STZ‐treated rats compared to ENDO (60.6 ± 1.3 ms, *n* = 122 cells, 20 hearts) myocytes from controls. There was also a modest prolongation of TPK in EPI myocytes from STZ‐treated compared to controls (Fig. 2C). THALF decay of the Ca^2+^ transient was significantly prolonged in EPI (189.6 ± 5.3 ms, *n* = 111 cells, 20 hearts) myocytes from control compared to ENDO (150.3 ± 3.4 ms, *n* = 122 cells, 20 hearts) myocytes from control rats. THALF decay was also significantly prolonged in ENDO (189.2 ± 5.3 ms, *n* = 113 cells, 23 hearts) myocytes from STZ‐treated rats compared to ENDO (150.3 ± 3.4 ms, *n* = 122 cells, 20 hearts) myocytes from controls (Fig. 2D). Amplitude of the Ca^2+^ transient was not significantly (*P* > 0.05) altered in EPI and ENDO myocytes from STZ‐treated rats or controls (Fig. 2E). In an attempt to clarify the mechanism(s) underlying these differences in Ca^2+^ transient, SR Ca^2+^ and L‐type Ca^2+^ current, the primary trigger for SR Ca^2+^ release, was investigated.

### Sarcoplasmic reticulum Ca^2+^

**Figure 3:**
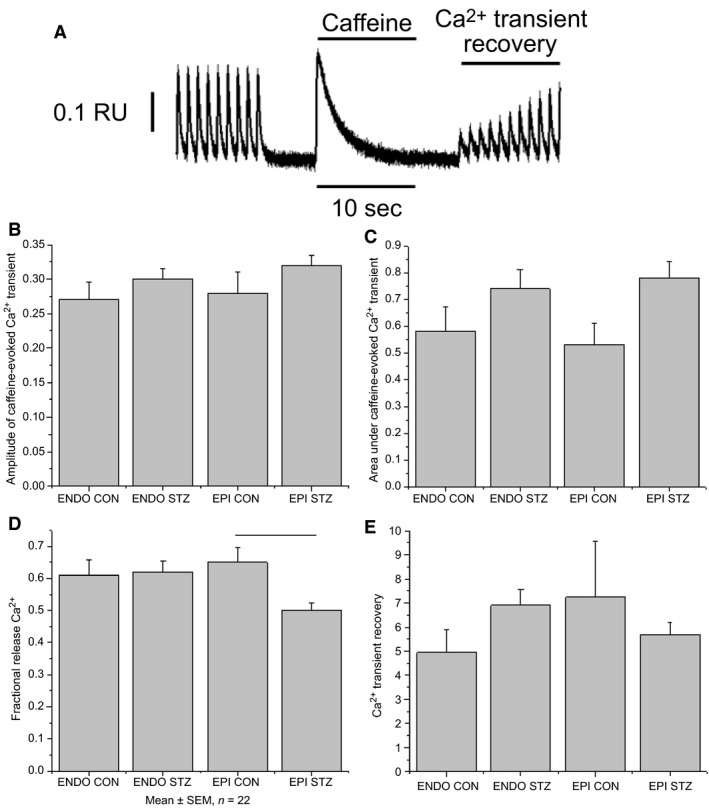
Sarcoplasmic reticulum Ca^2+^. Typical record showing protocol employed in a control ventricular myocyte during SR Ca^2+^ experiments (A). Initially Ca^2+^ transients were recorded during electrical stimulation. Electrical stimulation was then paused for 5 sec and 20 mmol/L caffeine was rapidly applied for 10 sec. After application of caffeine, electrical stimulation was resumed. Bar graphs showing mean amplitude of caffeine‐evoked Ca^2+^ transients (B), area under the curve of the caffeine‐evoked Ca^2+^ transient (C), fractional release of Ca^2+^, (D) and recovery of Ca^2+^ transients following rapid application of caffeine (E). Data are mean + SEM,* n* = 17–37 cells from 20 to 23 hearts. RU = Fura‐2 ratio units. Horizontal lines above the bars represent significant differences at the level of *P* < 0.05.

A typical record showing electrically evoked and caffeine‐evoked Ca^2+^ transients is shown in Figure 3A. Neither the amplitude of the caffeine‐evoked Ca^2+^ transient nor the area under the curve of the caffeine‐evoked Ca^2+^ transient were significantly altered in EPI and ENDO myocytes from STZ‐treated rats or controls (Fig. 3B and C). Fractional release was significantly reduced in EPI myocytes from STZ‐treated rats (0.49 ± 0.02, *n* = 37 cells) compared to controls (0.65 ± 0.05, *n* = 21 cells). Recovery of Ca^2+^ transients after application of caffeine and following resumption of electrical stimulation were not significantly altered in EPI and ENDO myocytes from STZ‐treated rats or controls (Fig. 3E).

### L‐type Ca^2+^ current

**Figure 4:**
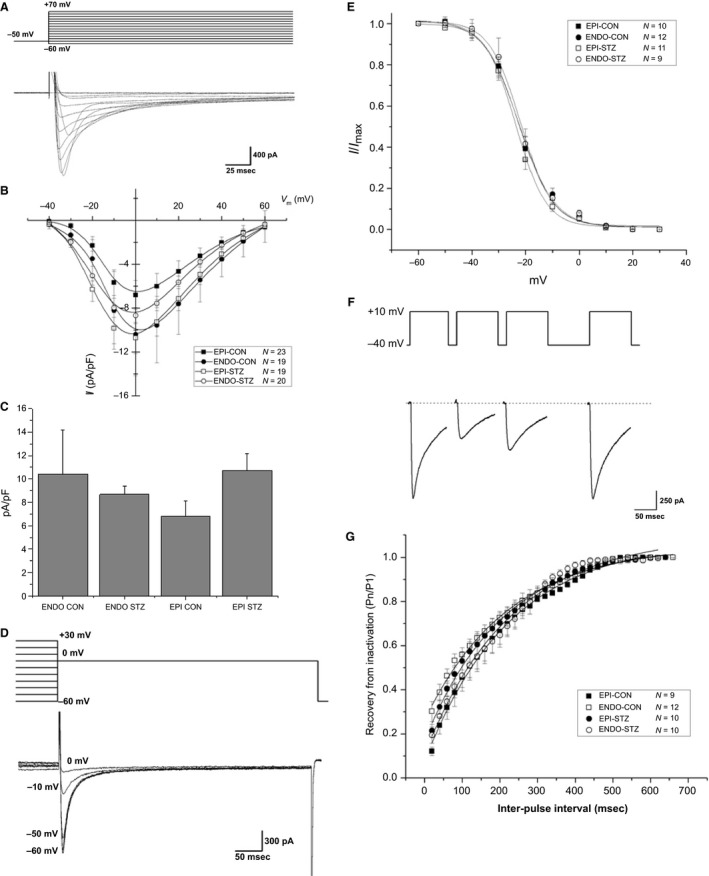
L‐type Ca^2+^ current. Typical records of *I*_Ca,L_ in a control cell are shown in (A). Graph showing amplitude of membrane currents evoked at different test potentials in the range −40 to +60 mV (B). Mean *I*_Ca,L_ evoked by test potentials to zero mV (C). Typical records of *I*_Ca,L_ inactivation in a control cell are shown in (D). Graph showing membrane currents following different prepulse potentials in the range −60 to +30 mV (E). Typical records of *I*_Ca,L_ during recovery from inactivation in a control cell are shown in (F). Graph showing recovery from inactivation at various interpulse intervals with variable duration (G). Data are mean + SEM,* n* = 9–12 cells from 9 to 11 hearts.

Typical recordings of *I*_Ca,L_, evoked at various test potentials, in a control cell are shown in Figure 4A. A graph of the current–voltage relationship is shown in Figure 4B. In the test range of −20 to +40 mV, *I*_Ca,L_ was largest in EPI myocytes from STZ‐treated rats and ENDO myocytes from controls, followed by ENDO myocytes from STZ‐treated, and smallest in EPI myocytes from controls. At 0 mV, the amplitude of *I*_Ca,L_ was not significantly altered in EPI and ENDO myocytes from STZ‐treated rats or controls (Fig. 4C). Typical recordings of *I*_Ca,L_ inactivation in a control cell are shown in Figure 4D. Inactivation was measured as the relationship between the amplitude of peak current at a test pulse of 0 mV and the amplitude of peak current during 1000‐ms long prepulses to different voltages between −60 and +30 mV. A graph of normalized peak currents following different prepulse potentials is shown in Figure 4E. Steady‐state inactivation was not significantly altered in EPI and ENDO myocytes from STZ‐treated rats or controls (Fig. 4E). Examples of typical records of *I*_Ca,L_ during recovery from inactivation in a control cell are shown in Figure 4F. Recovery from inactivation was investigated using a two‐pulse protocol, in which two 100‐ms depolarizing pulses to +10 mV were separated by interpulse intervals of variable duration. The peak Ca^2+^ current amplitude measured during the second pulse was normalized to the current measured by the first pulse and their ratio was plotted against the interpulse interval. A graph of normalized peak current ratios plotted against the interpulse interval is shown in Figure 4G. The time course of recovery from inactivation was not significantly altered in EPI and ENDO myocytes from STZ‐treated rats or controls (Fig. 4G).

## Discussion

The major findings of this study were: (1) TPK shortening was prolonged to similar extents in ENDO and EPI myocytes from STZ‐treated rats compared to ENDO and EPI myocytes from controls; (2) THALF relaxation of shortening was prolonged in ENDO myocytes from STZ‐treated rats compared to ENDO controls; (3) TPK Ca^2+^ transient was prolonged in ENDO myocytes from STZ‐treated rats compared to ENDO controls; (4) THALF decay of the Ca^2+^ transient was prolonged in ENDO myocytes from STZ‐treated rats compared to ENDO controls; (5) SR fractional release of Ca^2+^ was reduced in EPI myocytes from STZ‐treated rats compared to EPI controls; (6) *I*_Ca,L_ activation, inactivation, and recovery from inactivation were not significantly altered in EPI and ENDO myocytes from STZ‐treated rats or controls.

Resting cell length was similar in EPI and ENDO myocytes from STZ‐treated rats and controls. These results are consistent with previous studies that have variously demonstrated unchanged resting cell length, width, and the calculated cell volume in myocytes isolated from whole ventricle from STZ‐treated rats compared to controls (Howarth et al. 2000, 2002; Hamouda et al. 2015). However, differences have been reported in other regional cell measurements. For example, White et al. (1988) reported that the cross‐sectional area is greater in ENDO compared to EPI myocytes, while De Clerck et al. (1984) reported no significant difference between resting cell dimensions and sarcomere length in EPI and ENDO ventricular myocytes. TPK shortening was prolonged to similar extents in EPI and ENDO myocytes from STZ‐treated rats and controls. While there did not appear to be any regional effects on TPK shortening in this study, the prolongation of TPK shortening observed in EPI and ENDO myocytes from STZ‐treated compared to controls is a finding consistent with some previous studies in myocytes isolated from whole ventricle (Choi et al. 2002; Howarth et al. 2002, 2009). THALF relaxation of shortening was significantly prolonged in ENDO but not in EPI myocytes from STZ‐treated rats compared to controls, suggesting regional variation in the effects of STZ‐induced diabetes on the left ventricle. Previous studies in STZ‐treated rats have variously reported prolonged or unaltered THALF relaxation in myocytes from whole ventricle (Howarth et al. 2002, 2006; Shao et al. 2011). The amplitude of shortening was similar in EPI and ENDO myocytes from STZ‐treated rats and controls. Alterations in Ca^2+^ transport may partly underlie these differences in myocyte shortening. Consistent with resting cell length data, resting fura‐2 ratio was similar in EPI and ENDO myocytes from STZ‐treated rats and controls. TPK Ca^2+^ transient was similar in control EPI and ENDO myocytes. However, there was regional variation between EPI and ENDO myocytes from STZ‐treated compared to control rats. TPK Ca^2+^ transient was significantly prolonged in ENDO myocytes from STZ‐treated rats compared to controls, while there were no significant alterations in EPI myocytes from STZ‐treated rats compared to controls. Prolonged TPK Ca^2+^ transient has been previously reported in myocytes isolated from whole ventricle of STZ‐treated rats compared to controls (Howarth et al. 2002, 2009). This might be explained by the altered flux of Ca^2+^ through L‐type Ca^2+^ channel or release of Ca^2+^ from the SR. THALF decay of the Ca^2+^ transient was prolonged in EPI compared to ENDO myocytes from controls and was prolonged in ENDO myocytes from STZ‐treated rats compared to control. Previous studies have reported prolonged decay of the Ca^2+^ transient in myocytes isolated from whole ventricle of STZ‐treated rat which may be attributed to dysfunctional uptake of Ca^2+^ by the SR Ca^2+^ ATPase or efflux of Ca^2+^ via the Na^+^/Ca^2+^ exchange (Takeda et al. 1996; Ishikawa et al. 1999; Howarth et al. 2002; Bracken et al. 2006; Lacombe et al. 2007; Shao et al. 2011). Consistent with the shortening data, the amplitude of the Ca^2+^ transient was similar in EPI and ENDO myocytes from STZ‐treated rats and controls. These interesting results demonstrate regional differences in time course of the Ca^2+^ transient in healthy ventricle in addition to differences in decay that can be attributed to STZ‐induced diabetes.

Sarcoplasmic reticulum Ca^2+^ content was similar in EPI and ENDO myocytes from STZ‐treated rats and controls, as evidenced by the amplitude and area under the curve of the caffeine‐evoked Ca^2+^ transient and by the recovery of the Ca^2+^ transient following application of caffeine and resumption of electrical stimulation. Interestingly, fractional release of SR Ca^2+^ was reduced in EPI myocytes from STZ‐treated rats compared to controls which might indicate that, although caffeine‐evoked release of SR Ca^2+^ does not appear to be altered, there may be a reduction in the ability of *I*_Ca,L_ to trigger release of Ca^2+^ from the SR.

Voltage–current relationship, inactivation, and restitution were similar in EPI and ENDO myocytes from STZ‐treated rats and controls. A number of studies have shown that *I*_Ca,L_ appears to be similar in rat ventricular myocytes from ENDO compared to EPI regions (Bryant et al. 1999; Volk et al. 1999; Volk and Ehmke 2002). Some studies have demonstrated reduced amplitude of *I*_Ca,L_ while others have reported no change in *I*_Ca,L_ in myocytes from whole ventricle of STZ‐treated rats compared to controls (Choi et al. 2002; Bracken et al. 2006).

In conclusion, regional differences in Ca^2+^ transport may partly underlie differences in ventricular myocyte shortening across the wall of the healthy and the STZ‐treated rat left ventricle.

## Conflict of Interest

The authors have no conflict of interest to declare.
